# Image classification with symbolic hints using limited resources

**DOI:** 10.1371/journal.pone.0301360

**Published:** 2024-05-21

**Authors:** Mikkel Godsk Jørgensen, Lenka Tětková, Lars Kai Hansen

**Affiliations:** Department of Applied Mathematics and Computer Science, Technical University of Denmark, Kgs. Lyngby, Denmark; Mirpur University of Science and Technology, PAKISTAN

## Abstract

Typical machine learning classification benchmark problems often ignore the full input data structures present in real-world classification problems. Here we aim to represent additional information as “hints” for classification. We show that under a specific realistic conditional independence assumption, the hint information can be included by late fusion. In two experiments involving image classification with hints taking the form of text metadata, we demonstrate the feasibility and performance of the fusion scheme. We fuse the output of pre-trained image classifiers with the output of pre-trained text models. We show that calibration of the pre-trained models is crucial for the performance of the fused model. We compare the performance of the fusion scheme with a mid-level fusion scheme based on support vector machines and find that these two methods tend to perform quite similarly, albeit the late fusion scheme has only negligible computational costs.

## 1 Introduction

While the typical machine learning classification benchmark involves a single input measurement (say, an image in image classification), most real-world classification challenges involve more complex input data structures, often ignored in the benchmark data sets. In general, we hypothesize that such additional data may, in fact, be potential supporting information and, therefore, think of it as “hints”. Classification hints could simply be omnipresent image metadata (location, time, data etc.), information about the imaging process, or information relating to the image’s provenance (how the image transferred from capture to dataset). A straightforward application of the experiments carried out in this paper is a classification of images on the internet. One could enhance the performance by using text shown close to the image itself (supposing that the accompanying text is usually connected to the image).

Another application might be the quality control of grains: it is hard to detect certain diseases and damages based only on an image of a grain. However, one can use metadata about the production of the grains (e.g., the location of the field, weather conditions or how the grains were stored) as a hint that makes the task easier. Here we ask: How could we incorporate such high-level, symbolic information? A straightforward approach is to model adding hint information as an instance of data fusion. For fusion, we ‘embed’ the symbolic information, e.g. via a neural model, to produce a hint feature vector representation. The embedding corresponds to a separate data modality complementing the image.

Data fusion is typically carried out in the form of early fusion, where the feature vectors for the entering modalities are concatenated to allow for general dependency structures; as mid-level fusion, where modality-specific pre-processing steps are carried out in parallel, before feature vector concatenation; or as late fusion, where we process the inputs separately and combine the classification outputs. Here we show that under a specific and realistic conditional independence assumption, we can apply late fusion, providing a simple and fast fusion mechanism.

The late fusion scheme is derived from Bayes’ theorem in the ideal situation of having well-calibrated classifiers in combination with the assumption that the observed feature vectors are independent modalities given the class label. This assumption has been invoked earlier for data fusion, see e.g. [1, 2]. A similar assumption related to features rather than modalities is also a cornerstone in the naïve Bayes classifier (e.g. [3]).

Other more flexible fusion schemes include a (weighted) sum of the predicted probabilities (e.g. [4, 5]), or “Logarithmic Opinion Pooling” (e.g.: [4]).

In two experiments involving image classification with hints taking the form of text metadata, we demonstrate the feasibility and performance of the fusion scheme combining outputs of pre-trained unimodal classifiers. We compare its performance to a mid-level fusion scheme based on support vector machines and find that these two methods tend to perform quite similarly, albeit the late fusion scheme is, in comparison, almost ‘free’ computationally. We specifically investigate the role of classifier calibration and find that the Bayesian fusion scheme is significantly improved if we re-calibrate the classifiers prior to the combination.

**Fig 1:**
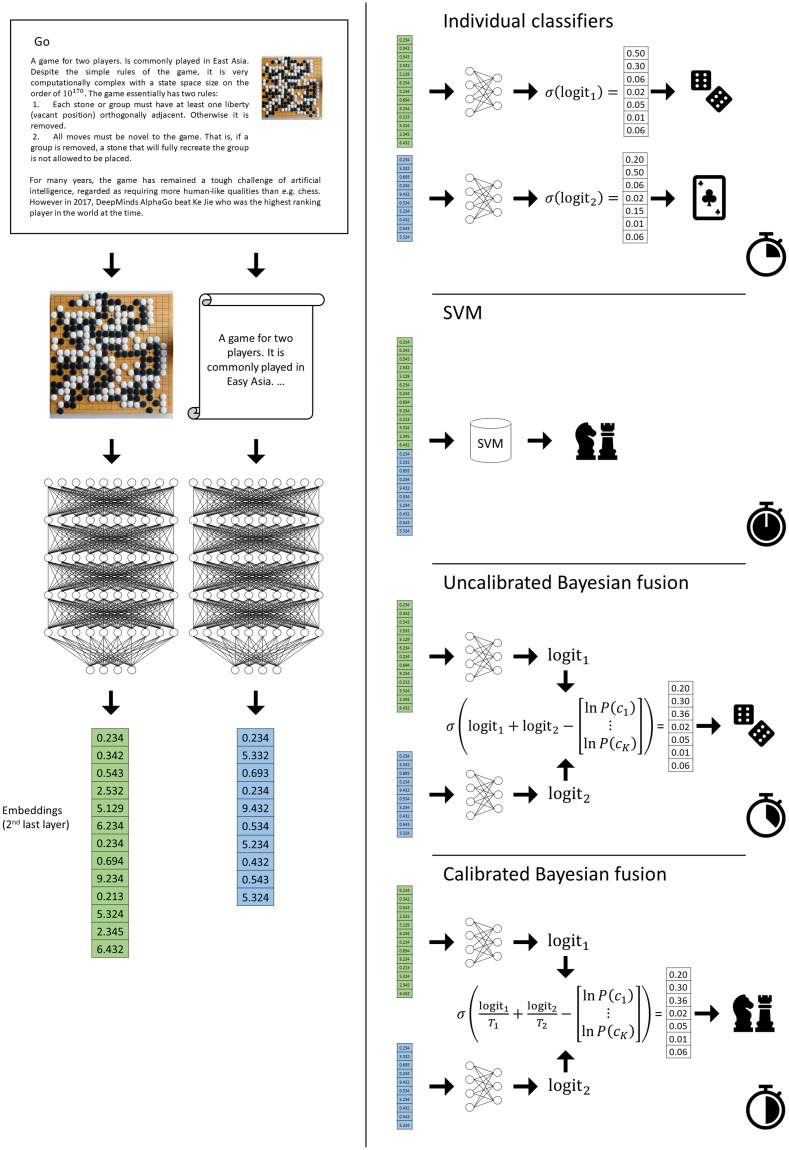
Summary of our approach. Summary of our approach to image classification with textual “hints”. An example of raw data is an image of a playing board and an article describing Go, the aim is to classify the image as a board game with the article used as a hint. On the left, we get embeddings for each modality independently. On the right, we show different approaches to model fusion studied in this paper. First, we build the individual constituent classifiers, which can perform classification in a reasonable time but with unsatisfactory accuracy. Next is the SVM mid-level fusion scheme, which is more accurate but very computationally expensive. Our contribution is two approaches to fusion—the uncalibrated and calibrated Bayesian fusion schemes, which offer a relatively inexpensive way of performing multi-modal classification. Calibration of the unimodal classifiers is critical for the performance of the fused model.

Our approach is visualized in Fig 1. The main contributions of this paper can be summarized as, we:

- formulate and prove Theorem 1 on Bayesian fusion of multiple classifiers;
- perform the empirical analysis of a late fusion scheme combining primary observation of interest with additional information provided in the form of hints at negligible cost;
- find that good calibration of the combined classifiers is critical for the fusion model.

### 1.1 Related work

There is a long history of combining separate pieces of information to improve the learning process and resulting models. In [6], Abu-Mostafa used hints in the form of prior knowledge about the unknown function to improve the model being trained, whereas we use additional information about a specific instance of input to improve its classification. There is a growing interest in including knowledge bases or metadata in the learning process for hybrid models combining neural networks with symbolic knowledge (e.g. [7, 8]).

There are also works focused on enhancing image classification with context metadata in various applications (e.g., [9–13]). In comparison, our approach makes use of already existing large pre-trained models eliminating the need for processing and incorporating the metadata into a complicated pipeline.

There are many approaches to combining multiple modalities [14–16]. Integration can happen at the input level (early fusion), at the decision level (late fusion) or intermediately [16]. Hybrid fusion [17] combines all the approaches. Like Axelsen et al. [1] and Chen et al. [2] derive a late Bayesian fusion scheme for integrating multiple visual classifiers with conditionally independent modalities, similar to our assumption. As noted, we are concerned with joining independent evidence in the form of hints of any type. Axelsen et al. [1] also propose a permutation test method to discover dependence among modalities in a dataset.

The efforts of combining vision and language have been explored in several works. Relevant tasks include visual question answering [18, 19] and visual reasoning [20, 21]. Models jointly trained on text and images have been developed (e.g. [22, 23]). These approaches typically solve more complex problems leading to intermediate-level data fusion.

## 2 Methods

Our method assumes existing models pre-trained for each modality independently. We build our fusion scheme on top of any deep learning classifiers, therefore, it can be used for any primary data and any type of hints. In our experiments, we focused on classifying images with the help of a single textual hint. In a case when a unimodal classifier is not available, we can create one by adding one or more linear layers on top of the embedding coming from a large-scale pre-trained model. Therefore, only training a linear layer is necessary. That is considerably faster and cheaper than training the whole classifier. An even more powerful classifier could be obtained by fine-tuning the whole pre-trained model. However, our focus is on the availability of the fused model, and fine-tuning of a large model is time- and resource-demanding.

### 2.1 Multimodal fusion by Bayesian inference

**Theorem 1**. *Given N observations* ***x***_1_, …, ***x***_*N*_ *and logits (i.e., outputs of the last linear layer, before the softmax function)* $z_{x_{1}},\ldots ,z_{x_{N}}$ *such that for all relevant i, j*: ${\text{softmax}}_{i}\left(z_{x_{j}}\right)\;=\;P\left(c_{i}|x_{j}\right)$, *and assume for all classes c*_*i*_ *that P*(***x***_1_, …, ***x***_*N*_, *c*_*i*_) > 0. *Then*

$$\begin{array}{c} \begin{array}{c} P\left(c_{i}|x_{1},\ldots ,x_{N}\right)={\text{softmax}}_{i}(\sum_{j=1}^{N}z_{x_{j}}+\text{ln}\;\kappa (x_{1},\ldots ,x_{N})-\left(N-1\right)\text{ln}\;\pi ), \end{array} \end{array}$$

*where* ***π*** *and* ***κ***(***x***_1_, …, ***x***_*N*_) *are vectors in* $R^{C}$ *with elements*

$$\begin{array}{c} \pi_{i}=P\left(c_{i}\right),\;\kappa_{i}(x_{1},\ldots ,x_{N})=\frac{P(x_{1},\ldots ,x_{N}|c_{i})}{P\left(x_{1}|c_{i}\right)\cdot \ldots \cdot P\left(x_{N}|c_{i}\right)}, \end{array}$$

*with C being the number of classes, and the logarithm is applied element-wise*.

*Remark*. Remind that $\text{softmax}:R^{N}\to R^{N}$ is defined as

$${\text{softmax}}_{i}\left(z\right)=\frac{{\text{exp}}^{z_{i}}}{\sum_{j=1}^{N}{\text{exp}}^{z_{j}}}\;\text{for}\;i\in 1,\ldots N\;\text{and}\;z\in R^{N}.$$

*Proof*. Using Bayes’ rule and multiplying by $\frac{\kappa_{i}\left(x_{1},\ldots ,x_{N}\right)}{\kappa_{i}\left(x_{1},\ldots ,x_{N}\right)}$, we obtain that:

$$\begin{array}{c} \begin{array}{cc} & P\left(c_{i}|x_{1},\ldots ,x_{N}\right)=\frac{P\left(x_{1},\ldots ,x_{N}|c_{i}\right)P\left(c_{i}\right)}{P(x_{1},\ldots ,x_{N})}\\ = & (\prod_{j=1}^{N}P\left(c_{i}|x_{j}\right))\frac{\kappa_{i}(x_{1},\ldots ,x_{N})}{\pi_{i}^{N-1}}\frac{\prod_{j=1}^{N}P\left(x_{j}\right)}{P(x_{1},\ldots ,x_{N})}. \end{array} \end{array}$$ (1)

We notice that the fraction $\frac{P(x_{1},\ldots ,x_{N})}{\prod_{j=1}^{N}P\left(x_{j}\right)}$ can be rewritten as such:

$$\begin{array}{c} \begin{array}{cc} & \frac{P(x_{1},\ldots ,x_{N})}{\prod_{j=1}^{N}P\left(x_{j}\right)}=\sum_{i=1}^{C}\frac{P\left(x_{1},\ldots ,x_{N}|c_{i}\right)P\left(c_{i}\right)}{P\left(x_{1}\right)\cdot \ldots \cdot P\left(x_{N}\right)}\\ = & \sum_{i=1}^{C}\frac{\kappa_{i}(x_{1},\ldots ,x_{N})}{\pi_{i}^{N-1}}\prod_{j=1}^{N}P\left(c_{i}|x_{j}\right)\\ = & \sum_{i=1}^{C}\frac{\kappa_{i}(x_{1},\ldots ,x_{N})}{\pi_{i}^{N-1}}\prod_{j=1}^{N}\frac{e^{z_{x_{j},i}}}{S(z_{x_{j}})}\\ = & \frac{S(\sum_{j=1}^{N}z_{x_{j}}+\text{ln}\;\kappa \left(x_{1},\ldots ,x_{N}\right)-\left(N-1\right)\;\text{ln}\;\pi )}{S\left(z_{x_{1}}\right)\cdot \ldots \cdot S\left(z_{x_{N}}\right)}, \end{array} \end{array}$$ (2)

where $S\left(z\right)=\sum_{i=1}^{C}e^{z_{i}}$. Now we substitute softmax for *P*(*c*_*i*_|***x***_*j*_) as well as the above result into Eq 1 to get:

$$\begin{array}{c} P\left(c_{i}|x_{1},\ldots ,x_{N}\right)={\text{softmax}}_{i}(\sum_{j=1}^{N}z_{x_{j}}+\text{ln}\;\kappa (x_{1},\ldots ,x_{N})-\left(N-1\right)\text{ln}\;\pi ). \end{array}$$

*Remark*. If we assume *P*(**x**_1_,…, ***x***_*N*_, *c*_*i*_) = 0 for some possible realization, then ln *κ*_*i*_(***x***_**1**_, …, ***x***_***N***_) or ln *π*_*i*_ is undefined and we have *P*(*c*_*i*_|***x***_**1**_, …, ***x***_***N***_) = 0.

If we assume that *P*(***x***_1_, …, ***x***_*N*_|*c*_*i*_) = P(***x***_1_|*c*_*i*_) · … · *P*(***x***_*N*_|*c*_*i*_), and avoid using *κ*_*i*_(***x***_**1**_, …, ***x***_***N***_) to resolve dependencies in the derivation, we get the same result as in Eq 3. Here we relax the assumption to *P*(*c*_*i*_) > 0.

*Remark*. We see that for ordinary logistic regression on the concatenated embeddings, a weight and a bias exist such that it is equal to the naive Bayes fusion (i.e. when ln *κ*(***x***_1_, …, ***x***_*N*_) = 0). Assume we have $z_{x_{j}}=W_{j}v_{x_{j}}+b_{j}$ for all *N* classifiers, as well as the block-matrices ***W*** = [***W***_1_| … |***W***_*N*_], and $v=[v_{x_{1}}^{T}\left|\ldots \right|v_{x_{N}}^{T}{]}^{T}$, and the bias $b=\left(1-N\right)\;\text{ln}\;\pi +\sum_{j=1}^{N}b_{j}$. We then see that

$$\text{softmax}(Wv+b)=\text{softmax}(\left(1-N\right)\text{ln}\;\pi +\sum_{j=1}^{N}z_{x_{j}}).$$

We notice that in the case of *N* = 2, the ln *κ*_*i*_ elements are the conditional mutual information of the observations conditioned on each class *i*. We further notice that for equiprobable classes, the (*N* − 1) ln ***π*** term can be left out since softmax is invariant to translation by any scalar multiple of the one-vector. The result generalizes the derivation in [1, 2] to include a term correcting for the naive Bayes assumption of independent modalities given class. If we suppose that modalities (observations and hints) are independent given class, we can simplify the result in Theorem 1 (***κ***(***x***_1_, …, ***x***_*N*_) = 1) and get

$$\begin{array}{c} \begin{array}{c} P\left(c_{i}|x_{1},\ldots ,x_{N}\right)={\text{softmax}}_{i}(\sum_{j=1}^{N}z_{x_{j}}-\left(N-1\right)\text{ln}\;\pi ). \end{array} \end{array}$$ (3)

We discuss conditional independence further in Section 2.3: Conditional independence. The vector ln ***π*** can in practice be computed by counting before performing any inference in the combined model.

Supposing we have a classifier of each modality, we can use this formula to estimate posterior probabilities by combining the logits and prior probabilities. This new model combines the original predictions (logits) with new information coming from processing hints. If the original classifiers are good, this combination generates better predictions.

### 2.2 Calibration

Since the result was derived using the posterior class probabilities, approximating these conditional probabilities well is crucial to using the model fusion approach in practice. However, as noted in [24], modern neural network classifiers are not guaranteed to be well-calibrated. For this reason, we quantify the effect of calibration of the classifiers on the accuracy of the fusion model. Since we add the logit vectors in our fusion scheme, their magnitudes play a major role—if the discrepancy is too large, one model will dominate the other in the decision. In our experiments, we used temperature scaling [24] to make the magnitudes reflect the accuracy of the model. We calibrated the models on their respective validation sets using 25 bins. We can summarize the approach of our calibrated Bayesian fusion (see Fig 1, bottom-right corner) in the following steps:

1. Get or create unimodal classifiers for the primary observation and all hint modalities.
2. Calibrate all the models.
3. Insert each modality of the input into its respective model and collect the logit vectors.
4. Sum all the logits, subtract prior probabilities and apply the softmax function on this vector (following Eq (3)).

We compare the performance of our late fusion model to an intermediate fusion scheme based on support vector machines. A linear SVM classifier [25] is trained on concatenated embeddings coming from each unimodal classifier. As embeddings, we take outputs of the second-to-last layer of the classifiers or the immediate outputs of the large-scale pre-trained models. They can vary in dimensions for each modality. We do it to compare the performance of our fusion model to what can be achieved with the embedding vectors as inputs.

### 2.3 Conditional independence

An important assumption of Eq (3) is the conditional independence of all modalities, conditioned on the class (label). In practice, this assumption is often satisfied. For example, imagine we have images of different objects and textual descriptions of the same types of objects. Once we know that the object is a chair, the images and the textual descriptions are independent.

When classifying MNIST images into two classes: even and odd, the independence condition is unfulfilled. A text describing a certain digit would reveal more information than just parity. However, we could repair the dependence if we divide the classes further into individual digits and condition on these classes. This can be done in general: even if the mutual independence of ***x***_1_|*c*, …, ***x***_*N*_|*c* is not possible, one can often further partition into a set of subclasses denoted $C_{c}^{\prime }$ such that ***x***_1_|*c*′, …, ***x***_*N*_|*c*′ are mutually independent for all $c^{\prime }\in C_{c}^{\prime }$. One could then build a model classifying into all partitions and use Theorem 1 because the subclasses would be conditionally independent. To obtain the original class probability, one would sum over the probabilities of its subclasses: $P\left(c|x_{1},\ldots ,x_{N}\right)=\sum_{c^{\prime }\in C_{c}^{\prime }}P\left(c^{\prime }|x_{1},\ldots ,x_{N}\right)$.

An example illustrating this partition could be a classification of images of places with an additional image hint (e.g. an image of the same place taken from a different angle). Imagine a class “park” and two pictures taken in the same park during winter. Since it snows in the winter, both images contain snow, whereas images taken during summer do not. Conditioning only on class is not enough in this case. However, if we condition on both class and season (“park during winter”), we get the independence and can use our scheme.

If we are able to identify all latent variables, then we can condition on these variables, get conditionally independent data and use Theorem 1.

## 3 Experiments

In our experiments, we focused on classifying images using textual hints. We used two datasets of images: Places205 [26] and ImageNet [27]. We used text descriptions from Cross-Modal Places [28] for the Places205 dataset. For ImageNet, we used our custom dataset of sentences from Wikipedia articles and WordNet [29] synset descriptions. To link Wikipedia articles to their respective classes, we used the links provided in [30]. We took only the first paragraph of each article because it should contain the most relevant information related to the class. The texts from Wikipedia were split up into individual sentences and combined with WordNet glosses. Thus, each hint for ImageNet images is one sentence, either from the corresponding Wikipedia article or the WordNet synset. Since we were unable to find a Wikipedia description of some of the synsets, we discarded 9 classes from the dataset.

Both image datasets and CMPlaces text descriptions do not have publicly available test sets. Therefore, we used the validation sets for testing and split the training sets into training split and validation split in a ratio of 80% to 20%. For the sentences from Wikipedia and WordNet, we first set aside 20% for testing and then subdivided the training set similarly to the other datasets. Note that for all datasets, the training and validation splits were used for hyperparameter tuning but later merged to train the final model.

Textual hints are not linked to particular images. During training and evaluation, a textual hint from the corresponding class was randomly selected for each image. As a consequence, conditional independence is satisfied.

For the text classifier, we obtained the cased versions of BERT-768 (BERT-base) and BERT-1024 (BERT-large) from Huggingface [31, 32]. To build a text-classifier using BERT, we used the so-called *Average Word Embedding*, in which we averaged the hidden vectors corresponding to the input tokens (not including the next-sentence predictor, i.e. the [CLS] token). The reason for taking this approach was that Jørgensen found evidence for this being a more useful sentence-embedding vector than the next-sentence predictor in [33], although it has also received some criticism (see e. g. [34]). It is a 768d vector in the case of BERT-base, and a 1024d vector in BERT-large. We attached one linear layer with the softmax activation function to get a text classifier. For the experiments on CMPlaces, we used VGG16 [35] pre-trained on Places205 [36] for the image modality. For the experiments on ImageNet, we used ResNet50 [37] as offered through torchvision [38].

We conducted the following battery of experiments on both datasets. We:

1. trained a BERT-based classifier on the text modality and evaluated both the text and image classifier on their respective modalities to get unimodal baseline performances (see Fig 1, top-right corner);
2. combined the two uncalibrated classifiers using Eq (3) and evaluated the fusion model (Fig 1, third from top on right);
3. calibrated both classifiers and evaluated them. This experiment was motivated by Section 2.2: Calibration and our hypothesis that the fusion model performs better if we use well-calibrated unimodal classifiers as the base models;
4. combined the calibrated classifiers using Eq (3) and evaluated the fusion model (Fig 1, bottom-right corner);
5. trained a linear SVM classifier on the concatenated embeddings coming from both classifiers (i.e. for this experiment, we stripped the models of their classification heads) and evaluated it. The regularization parameter was tuned using the hold-out method (on a validation split of the training set) This is illustrated in Fig 1, second from top on right.

### 3.1 Training details

We used the Adam optimizer and the cross-entropy loss

$$\ell \left(y,p\right)=-\sum_{c=1}^{N}y_{c}\;\text{log}\;\left(p_{c}\right).$$

The metrics we used to compare our models are the top-1 and top-5 accuracies (i.e., proportion of the data such that the target label is within the 1 (or 5) highest-scoring predicted class(es)). In the tables, we used the conventional notation of displaying the accuracies in percentages. We used the 95% Jeffreys intervals to quantify uncertainty for the accuracies. Since the text modalities have fewer observations than the image modalities, we based the interval radii for the fusion models on the text only. This approach is likely overestimating uncertainty.

For CMPlaces, we used 20500 observations for the image models and 2050 observations for text/fusion. Similarly, we used 49550 and 1708 for ImageNet, in the same order.

We used Scikit-Learn [39] to implement the SVM classifier. To tune the hyperparameters of the text classifier, we used the TPESampler (Tree-structured Parzen Estimator) procedure in Optuna [40]. Here we optimized for top-1 accuracy in the text-classification task alone. We experimented both with using weight decay and dropout for regularizing the classification head. Also, we tuned the learning rate and the amount of layers (and hidden units) at the top part of the network. Since we found the best performance using only dropout, a linear layer, and a tuned learning rate, we tuned these exclusively in the end. Throughout the search, we tuned the model on a training split (80%) for 5 epochs and validated on a validation split (20%). The splits were picked randomly at the beginning of the tuning and were fixed across all trials.

**Fig 2:**
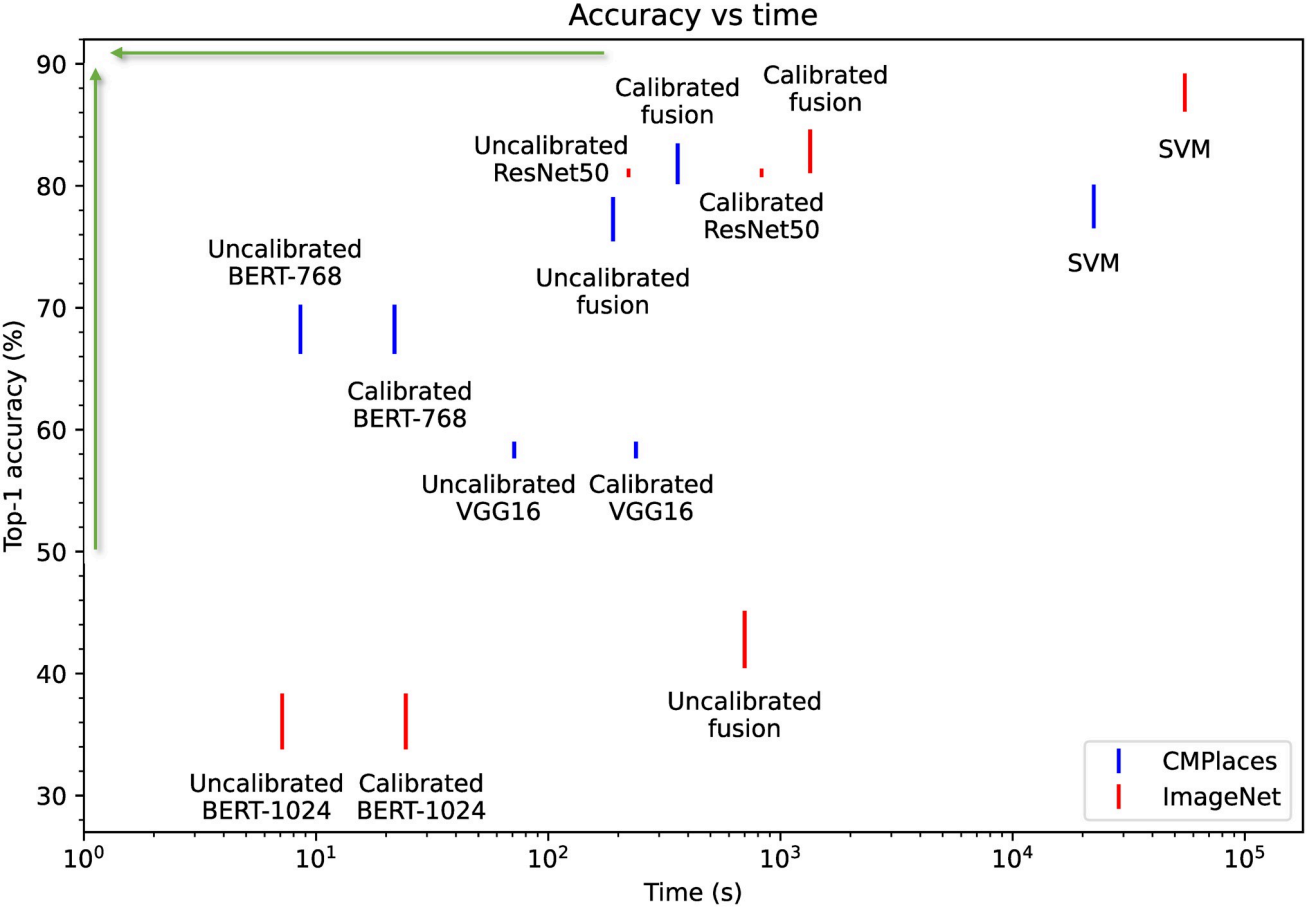
Accuracies vs time. The top-1 accuracies of each model plotted with uncertainties against the time spent on preparing and evaluating them. The best models are in the upper left corner as indicated by the green arrows. Here we suppose that we are provided with trained constituent classifiers. Hence, for the Bayesian fusion models, the preparation time only includes the time to calibrate and estimate the log-prior. The preparation time of SVM comprises training and calibrating the SVM head.

We tuned the regularization strength in the SVM-based fusion scheme manually on the training split and validated it on the validation split. We optimized for the best top-1 accuracy in the combined task. We presented a large spectrum of regularization values and tested the performance after training for 1 epoch. We chose 1 epoch since it gave a good performance but was also very time-demanding. Note here that the training split is comparable in size to the size of the entire training set, which proved to be very time-consuming to train on (see Fig 2).

In order to calibrate the image and text classifiers, we used LBFGS with a fixed learning rate. We used the implementation from [41] which can be found on the associated GitHub repository. The calibrations of the image and text classifiers were done on the validation sets of the image and text modalities respectively. We find this choice justifiable since calibration does not affect the predictions of the models individually, but only affects their confidence in prediction. Furthermore, the calibration aligns the prediction confidence of the model with the prediction accuracy. And since we want the model to be as close as possible to predicting the true conditional probability *p*(*c*|***x***), it makes sense to calibrate it on the validation set assuming it is representative enough of the true underlying distribution.

The implementations and settings of the searches are all found in the associated GitHub repository of this paper [42]. The selected hyperparameters are found set in the code in the file main.py to make the experiments reproducible.

### 3.2 Results

Table 1 summarizes the results of the experiments described in the previous part. In both experiments, we see that fusing calibrated models produced a significant improvement over each of the two constituent classifiers, as well as the uncalibrated fusion model.

**Table 1:** Results of the experiments.

a) Cross-Modal Places	Top-5 (%) ↑	Top-1 (%) ↑	ECE ↓	MCE ↓
Uncalibrated BERT-768	88 ± 1	68 ± 2	24.86	50.93
Uncalibrated VGG16	86.6 ± 0.5	58.3 ± 0.7	2.87	6.79
Uncalibrated fusion	92 ± 1	77 ± 2	18.54	47.31
Calibrated BERT-768	88 ± 1	68 ± 2	6.21	13.78
Calibrated VGG16	86.6 ± 0.5	58.3 ± 0.7	**2.01**	**5.97**
Calibrated fusion	**96.7** ± **0.8**	**82** ± **2**	5.48	12.28
SVM	94.3 ± 1.0	78 ± 2	10.59	20.09
b) ImageNet				
Uncalibrated BERT-1024	54 ± 2	36 ± 2	51.19	75.73
Uncalibrated ResNet50	95.5 ± 0.2	81.1 ± 0.3	53.46	62.95
Uncalibrated fusion	58 ± 2	43 ± 2	45.80	67.88
Calibrated BERT-1024	54 ± 2	36 ± 2	4.88	19.07
Calibrated ResNet50	95.5 ± 0.2	81.1 ± 0.3	**2.97**	27.34
Calibrated fusion	**96.9** ± **0.8**	83 ± 2	6.46	**13.83**
SVM	**97.7** ± **0.7**	**88** ± **2**	8.13	15.51

Results of the experiments on validation sets of a) Places205 with text hints from CMPlaces and b) ImageNet with Wikipedia/WordNet hints. For calibration, we estimated the *Expected Calibration Error* (ECE) and the *Maximum Calibration Error* (MCE) as described in [24]. We deliberately worsened the calibrations of the unimodal classifiers (Uncalibrated BERT-768, Uncalibrated VGG16, Uncalibrated BERT-1024 and Uncalibrated ResNet50) to investigate the effect of using well-calibrated models.

We broke the calibrations of the unimodal classifiers by temperature scaling to observe their importance on the results of our fusion scheme. If badly-calibrated models are used, the logits may be of different magnitudes, and one of the classifiers may outweigh the other. Logits with comparable magnitudes are needed for a balanced voting process. Our findings agree with the observation Chen et al. made in [2] that calibration is crucial for this type of model fusion.

Calibrating the unimodal classifiers is important not only for classification accuracy but also for getting a well-calibrated fusion model. Our results indicate that fusing uncalibrated models yields an uncalibrated fusion model, and fusing well-calibrated unimodal classifiers results in a well-calibrated fusion model.

We can also observe that the calibrated fusion models have better or comparable performance to the linear SVM classifiers. It tells us that we cannot get substantially better models built on top of the embedding vectors.

We report the time needed to conduct the experiments in Fig 2. It shows that SVMs require considerably more time and resources to perform well than calibrated fusion models. This indicates that our fusion scheme can give a roughly equal success rate with only negligible resource requirements.

## 4 Conclusion

In this paper, we proposed a late fusion scheme for object classification using additional knowledge in the form of “hints”. We showed that we could improve the performance by combining pre-existing unimodal classifiers. When compared to a linear SVM classifier, our fusion model achieved comparable accuracy with much less computational resources. We also revealed that calibration of the unimodal classifiers is crucial for the performance of the fusion model. Future work could explore other ways of combining primary observations with additional knowledge to further improve performance.
